# Using high titer West Nile intravenous immunoglobulin from selected Israeli donors for treatment of West Nile virus infection

**DOI:** 10.1186/1471-2334-9-18

**Published:** 2009-02-17

**Authors:** David Ben-Nathan, Orly Gershoni-Yahalom, Itzchak Samina, Yevgeny Khinich, Israel Nur, Orgad Laub, Ahuva Gottreich, Michael Simanov, Angel Porgador, Bracha Rager-Zisman, Nadav Orr

**Affiliations:** 1 The Shraga Segal Dept. of Microbiology and Immunology, Ben Gurion University, Beer Sheva, Israel; 2 Kimron Veterinary Institute, Department of Virology, Beit Dagan, Israel; 3 OMRIX Biopharmaceuticals, Weizmann Science Park, Ness-Ziona, Israel; 4 MDA National Blood Services, Tel Hashomer, Kiryat Ono, Israel

## Abstract

**Background:**

West Nile Virus (WNV) is endemic in Israel and a significant level of antibodies is present in the population due to natural exposure. Anecdotal cases suggested that the presence of anti-WNV antibodies in intravenous immunoglobulin (IVIG) from Israeli donors (IVIG-IL) assisted the recovery of patients with severe WNV infection.

**Methods:**

To enhance the therapeutic efficacy of IVIG-IL against WNV infection, OMRIX Biopharmaceuticals, Israel, have developed a strategy for selection of plasma units from a 10% fraction of Israeli blood donors with anti-WNV antibodies. Positive units were processed into pharmaceutical grade WNV IVIG (WNIG). Following inoculation with WNV, mice received i.p. injections of different doses (0.01–8 mg/mouse) of IVIG-IL or WNIG, according to the specific experimental protocol.

**Results:**

WNIG was about 10 times more potent (per gr of IgG) than was regular IVIG-IL when tested by ELISA and neutralization assays. In a mouse lethal WNV infection model, prophylactic treatment with WNIG was at least 5–10-fold more potent as compared to treatment with IVIG-IL. Treatment with WNIG during active encephalitis, three or four days following WNV infection, had a significant protective effect. WNIG was also very effective in protecting immunosuppressed mice. Indeed, treatment of dexamethasone-immunosuppressed mice with 0.2 or 1.0 mg WNIG 4 h after virus infection, led to 100% survival.

**Conclusion:**

IVIG produced from selected plasma donated in WNV endemic regions can be used to produce WNV IVIG with superior activity for therapeutic and prophylactic measures.

## Background

Passive transfer of antibodies has been shown to be effective for the prevention and treatment of many infectious diseases, including those caused by viruses [1]. Intravenous human immunoglobulin produced from pooled plasma (IVIG) is the major source for antibody therapy by virtue of the diverse repertoire of immunoglobulin molecules responsible for a wide spectrum of anti-bacterial and anti-viral activities [2]. The pooled plasma of subjects that had been naturally exposed to pathogens has been used for the production of IVIG preparations containing specific antibodies for treatment of disease causing viruses, including Cytomegalovirus, Hepatitis A, B and C, HIV, Respiratory Syncytial Virus, Measles and Varicella Zoster virus [1].

West Nile virus (WNV), a mosquito-transmitted flavivirus, was first isolated from a febrile adult woman in the West Nile District of Uganda in 1937 [3]. WNV is a single stranded plus RNA virus, and a member of the Japanese encephalitis antigenic complex of the genus *Flavivirus*, family *Flaviviridae *[4,5]. Until 1999, West Nile Virus was found in Africa, the Middle East, parts of Asia, Southern Europe and Australia. It then suddenly emerged in New York, rapidly spread throughout the United States and has since caused considerably acute mortality and morbidity [6]. The clinical manifestations of WNV in humans range from asymptomatic seroconversion to fatal meningo-encephalitis, with symptoms including cognitive dysfunction, muscle weakness and flaccid paralysis [7-10]. Depressed immunity, age and genetic factors [11,12] are correlated with greater risk for neurological disease. Currently, there is no effective anti-viral therapy or human vaccine for WNV infection.

Available evidence suggests that WNV might be more susceptible to antibody-mediated than cell-mediated immunity. Indeed, passive transfer of specific antibodies (Ab) or immunoglobulin has been shown to abort or modify West Nile viral infections in animal models in a dose-dependent manner [13-15]. WNV is endemic in Israel and significant levels of anti-WNV Ab are found in commercial preparations of IVIG prepared from the plasma of Israeli donors (IVIG-IL). Anecdotal cases have suggested that the presence of anti-WNV Ab in IVIG-IL assisted the recovery of patients suffering from severe WNV infection [16,17]. We have previously shown that while IVIG-IL protected mice against lethal doses of WNV, the low exposure to the virus of US donors resulted in no effect of IVIG produced from the plasma of US donors (IVIG-US) [13,18]. Recently, however, it has been shown that some IVIG preparations produced in the USA during epidemic years contained antibodies against WNV and thus, were protective in an animal model for WNV infection [19].

In order to enhance the therapeutic efficacy of IVIG against WNV infection, OMRIX biopharmaceuticals firm has developed a strategy for the selection of plasma units from the 10% fraction of blood donors containing WNV antibodies. Positive units were processed into pharmaceutical grade WNV IVIG (WNIG). The potency of WNIG for the neutralization of WNV NY99 strain was tested *in vitro *by a cell neutralizing assay and *in vivo*, using a mouse lethal model. WNIG was at least 5–10-fold more potent than was regular IVIG-IL. Treatment with WNIG three or four days after challenge was also efficacious. We conclude that blood from selected donors in WNV endemic regions can improve the potency of IVIG and should be developed for use in therapy and for prophylactic measures.

## Methods

### Mice

Female BALB/cOlaHsd mice (4–5 weeks old, 15–17 g at study initiation; Harlan Laboratories, Israel) were used, unless otherwise stated. Mice were maintained in isolation cages throughout the study and fed and watered *ad libitum*. The mouse experiments were approved and performed according to the Kimron Veterinary Institute guidelines for animal experimentation with arboviruses.

### Cell Cultures

Vero cells (ATCC #CRL-1587) were grown in Dulbecco's Modified Eagle Medium (DMEM) supplemented with 10% fetal calf serum (FCS), 1% nonessential amino acids, 1.2% NaHCO_3 _and antibiotics. The cells were maintained in a humidified atmosphere at 37°C containing 5% CO_2 _and were used for growing virus stocks and virus titration.

### Virus stocks and virus titrations

WNV-NY99 (3rd passage in Vero cells of strain NY 385-99) [20], was kindly provided to OMRIX biopharmaceuticals by M.S. Diamond, Washington University, School of Medicine, St. Louis, MO, USA. Virus plaque assays were performed on Vero cells as previously described [21] and virus titers were expressed as plaque-forming units (PFU)/ml. A single virus stock containing 5 × 10^8 ^PFU/ml was stored in aliquots at -70°C and was used in all studies.

### Infection and challenge of mice

In all experiments (unless otherwise stated), 4–5 week old mice were inoculated intraperitoneally (i.p.) with 0.2 ml of coctail containing different quantities of stock virus per mouse [18]. Lethal dose 50 (LD_50_) was calculated according to the method of Reed and Muench [22].

### Immunoglobulin (IVIG) preparations

OMRIX IVIG preparations (Omr-IgG-am™, OMRIX biopharmaceuticals, Israel) contain 5% protein, consisting more than 99% IgG and a very small quantities of IgA and IgM. Three preparations (5% IVIG, produced by OMRIX) were employed (Table 1): (a) High titer WNV-IVIG (WNIG), Batch #K44G511. This product was prepared during 2006 from Israeli plasma that was pre-screened by ELISA for specific anti-WNV antibodies. About 10% of the plasma units from the general blood donor population had > 100 arbitrary units/ml (AU/ml) of anti-WNV antibodies. These units were pooled for the production of WNIG. (b) Commercial IVIG-IL batch #H23131, prepared in 2003, from pooled plasma of Israeli blood donors. (c) Commercial IVIG-US Batch #K30G370, prepared in 2006 from pooled plasma of US blood donors.

**Table 1:** Anti-WNV antibodies in the IVIG preparations used for treatments

	Titer by ELISA		Titer by PRNT_50_	
		
IVIG Preparation	AU/ml	AU/mg	WNU/ml	WNU/mg
WNIG	7608	152	9900	198
IVIG-IL	668	13	1032	21
IVIG-US	179	4	Not done	

WNIG, high titer anti-WNV IVIG (from selected donors); IVIG-IL, IVIG preparation from the pooled plasma of Israeli donors; IVIG-US, IVIG preparation from the pooled plasma of US donors. AU, arbitrary units of anti-WNV antibodies per mouse (as assessed by ELISA, see Methods). WNU, the reciprocal of the highest dilution giving 50% reduction in a PRNT_50 _assay.

### Antigen preparation

Inactivated WNV antigen was prepared by inactivation of the stock virus with *β*-Propiolactone (0.001%final concentration) [18,23].

### Detection of anti-WNV antibodies by ELISA

#### a. ELISA for human antibodies

A quantitative ELISA was developed at OMRIX based on the method described earlier [18]. To allow quantitative measurements, a specific positive plasma sample was assigned a value of 2000 AU/ml WNV antibodies. This sample served as a calibration standard. The 1–10 AU/ml range was found to display linear characteristics when the log-transformation of concentration was plotted against the observed optical density at 405 nm. Briefly, microtiter plates were first coated with inactivated WNV antigen in carbonate buffer, pH 9.6. The plates were blocked with 0.5% I-block (Tropix, Bedford, MA) and 10% goat serum. Samples and controls were diluted in blocking buffer to fit the standard curve range. Alkaline phosphatase-conjugated goat anti-human IgG (Sigma, Israel) was added as a detector followed by pNPP substrate (Sigma, Israel). The titer of each serum and control sample was calculated from absorbance at 405 nm plotted against the log transformation of calibrator concentration.

#### b. ELISA for mouse sera

An ELISA test was performed according to the method described by Martin et al. [24], with slight modifications [18]. Specific titers were expressed as the reciprocal of the highest dilution giving a reading above the cut-off value [25].

### Plaque Reduction Neutralization Titer 50% (PRNT50)

The titer of neutralizing antibodies was determined using a plaque reduction test based on the method described earlier [18]. Briefly, samples were diluted by serial two-fold dilutions (1:10 – 1:10,240) in DMEM, 2% FCS and mixed with an equal volume of similar medium containing approx. 450 PFU/ml of WNV NY99. The mixtures were incubated overnight at 4°C on a roller and the virus-antibody mixtures (400 μl) were then added to Vero cells pre-seeded in 6-well plates at a concentration of 5 × 10^5 ^cells/well and pre-grown overnight at 37°C, 5% CO_2_. After 60 minutes at 37°C in 5% CO_2_, the monolayers were overlaid with 3 ml of Modified Eagle Medium (MEME) ×2 and tragacanth (Sigma, Israel) containing 4% FCS and 2.4% sodium bicarbonate. The cultures were incubated for an additional 72 hours at 37°C in 5% CO_2_. The cells were then fixed with ethanol, stained with fucsin, and plaques were counted. PRNT_50 _values were expressed as the reciprocal of the highest dilution giving 50% reduction in plaque numbers (WNU).

### Treatment with Immunoglobulins

Following inoculation with WNV, mice received i.p. injections of different doses of IVIG-IL, IVIG-US or WNIG, according to the specific experimental protocol. Treatment doses ranged from 0.01 mg/mouse (about 0.6 mg/kg) to 8 mg/mouse (about 470 mg/kg). The animals were followed for mortality for at least 21 days.

### WNIG Therapy of immunosuppressed mice

Seven to 8 weeks old mice received two subcutaneous (s.c.) doses of dexamethasone (Sigma, Israel, 60 μg/mouse each). The first dose was administered 2 hours before inoculation with 5–10 PFU of WNV-NY99 and a second dose was administered 1 day thereafter. Four hours after inoculation with WNV, the animals were treated i.p. with 1 mg or 0.2 mg/mouse of WNIG. The animals were followed for mortality for at least 21 days.

### Statistics

Survival rates of the different groups were compared by Fisher's exact test. Average days for death were compared by Student's t-test.

## Results

### The protective effects of WNIG against lethal WNV infection

To study the relative protective efficacy of WNIG, as compared to IVIG-IL or IVIG-US, mice were inoculated i.p. with 10–20 LD_50 _(50–100 PFU) of WNV-NY99, followed 4 hours later by treatment with a single injection of 2, 0.5 or 0.1 mg of WNIG, IVIG-IL or IVIG-US. Treatment with 2 mg of WNIG/mouse or IVIG-IL was sufficient to confer 88–100% protection against 10–20 LD_50 _of the virus (Table 2). As expected, IVIG-US showed lower protective efficacy, with only 63% survival after treatment with 2.0 mg/mouse. The superiority of WNIG was clearly demonstrated at a dose of 0.1 mg/mouse, where IVIG-IL conferred only 44% protection, as compared to the 94% protection attained with WNIG.

**Table 2:** Protective efficacy of WNIG, IVIG-IL and IVIG-US in mice infected with 10–20 LD_50 _of WNV NY99

Treatment (mg/mouse)	Specific Ab dose^a^	Percent survival (live/treated)
No treatment	0	7 (1/14)^b^
WNIG (0.1 mg)	15	94 (15/16)^c^
IVIG-IL (0.1 mg)	1.3	44 (7/16)
WNIG (0.5 mg)	76	100 (16/16)
IVIG-IL (0.5 mg)	7	87 (14/16)
WNIG (2.0 mg)	304	100 (8/8)
IVIG-IL (2.0 mg)	27	88 (7/8)
IVIG-US (2.0 mg)	7	63 (5/8)

Groups of 4–5 week-old BALB/c mice were treated i.p. with different amounts of WNIG or IVIG-IL, 4 h after injection of 10–20 LD_50 _of West Nile virus. Animals were observed daily for mortality for 21 days. ^a^Arbitrary units of anti-WNV antibodies per mouse (as assessed by ELISA, see Methods). ^b^p < 0.039, as compared to all treatment groups. ^c^p = 0.006, as compared to the IVIG-IL (0.1 mg) group.

These results suggest that the protective efficacy of 0.1 mg WNIG was similar to that obtained with 0.5 mg of IVIG-IL, i.e. WNIG was at least 5-fold more efficacious. WNIG at 0.1 mg showed a similar efficacy as IVIG-US at 2.0 mg, i.e. WNIG was some 20-fold more efficacious. The superiority of WNIG was also demonstrated by comparing the levels of viremia in mice treated with IVIG-IL or WNIG before inoculation with 50 PFU of WNV. Treatment with 0.1 mg IVIG-IL moderately decreased virus levels in the blood of infected animals from an average of 3.4 to 2.0 log_10 _PFU/ml on day 2 and from 2.9 to 1.8 log_10 _PFU/ml on day 3. No virus was detected in the blood of mice treated with WNIG. About 6.2 log_10 _PFU/brain were found on day **7 **in brains of control-infected mice, while no virus was detected in the brains of IVIG-IL- or WNIG-treated mice (data not shown).

**Figure 1:**
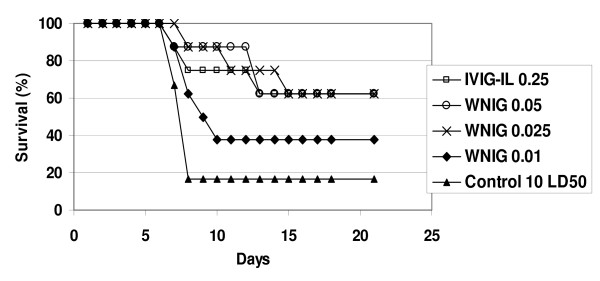
**Dose-dependent protection by WNIG**. Groups of 5-week old BALB/c mice were treated i.p. with IVIG-IL (0.5 or 0.25 mg/mouse) or with WNIG (0.01, 0.05, or 0.025 mg/mouse) 4 h after infection with 10 LD_50 _of WNV NY99. Mice were observed for mortality for 21 days. One group of infected mice received no treatment (control).

We further investigated the protective efficacy of lower doses of WNIG and IVIG-IL. Groups of mice were infected with 10 LD_50 _(50 PFU) of WNV and treated 4 hours later with a single injection containing 0.05, 0.025 or 0.01 mg of WNIG or 0.25 mg of IVIG-IL. As shown in Figure 1, a 5–10 fold increase in protective efficacy of WNIG over IVIG-IL treatment was maintained, as revealed upon comparing treatment with 0.25 mg IVIG-IL to treatment with 0.05–0.025 mg WNIG. Mortality in these groups was delayed compared to that of the control-infected mice. In the untreated WNV-NY99 infected mice, all of the deaths occurred by day 8. In the IVIG-IL (0.25 mg)-treated mice, mortality was delayed and occurred between days 8–15. A similar pattern of delayed mortality was obtained after treatment with 0.05 or 0.025 mg of WNIG.

#### Detection of anti-WNV antibodies in surviving mice

De-novo synthesis of anti-WNV antibodies in WNIG-treated, surviving mice was tested 21, 40 and 60 days after challenge and treatment. Animals were challenged with 10 LD_50_/mouse and 4 h later, were treated with WNIG or IVIG-IL (0.1–0.5 mg/mouse). Pooled serum samples were collected from surviving mice on days 21, 40 and 60 after treatment and antibody titers were assessed by ELISA. The surviving animals presented high levels of anti-WNV antibody titers ranging from 1:8,000–1:16,000. The high titers of anti-WNV antibodies were maintained for at least 60 days.

#### Therapeutic efficacy of WNIG

**Figure 2:**
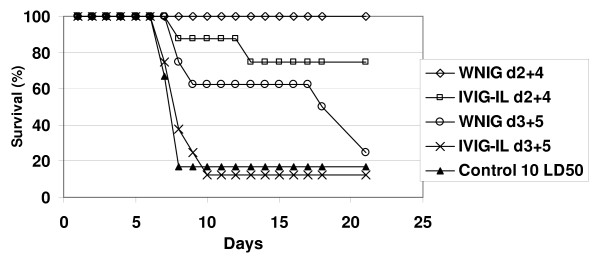
**Therapeutic efficacy of WNIG**. Groups of 5-week old BALB/c mice were treated i.p. with 2 mg/mouse IVIG-IL or WNIG on days 2 and 4, or days 3 and 5 after infection with 10 LD_50 _of West Nile virus. Mice were observed for mortality for 21 days. One group of infected mice received no treatment (control).

In the mouse model, active encephalitis can be detected 3 days after challenge with WNV [18,32]. To study the therapeutic efficacy of WNIG, mice were injected with 10 LD_50 _of WNV, and treated with 2 mg WNIG or IVIG-IL on days 2 and 4 or 3 and 5 after infection. As shown in Figure 2, injection of 2 mg of WNIG on days 2 and 4 after infection protected 100% of the animals, as compared with the 75% protection observed in mice receiving IVIG-IL. Treatment with WNIG on days 3 and 5 significantly delayed mortality of the infected mice. In this group, 62% of the animals survived for 17 days. The average period of death in the WNIG group treated on days 3 and 5 was 12.5 days (SD = 5.7), while the average period of death in the IVIG-IL group treated 3 and 5 days after infection and in untreated mice were 7.1 days (SD = 1.1) and 6.6 days (SD = 0.5), respectively (p = 0.032 for the WNIG group as compared to the IVIG-IL group; p = 0.048 for the WNIG group as compared to untreated group).

To assess the therapeutic effect offered by a higher dose of WNIG, a single dose of 8 mg/mouse was given on days 3, 4, or 5 after infection. This dosage protected all of the animals when administered 3 days after infection (Table 3). When given 4 days after infection, the survival rate was 63% as compared to the 25% survival rate measured in the untreated control group. Treatment on day 5 after infection was not effective, suggesting that, at this point, the damage cause by the virus was irreversible.

**Table 3:** Protective efficacy of WNIG in mice infected with 10 LD_50 _of WNV.

Treatment day (after challenge)	Treatment dose (mg/mouse)	Percent survival (live/treated)
No treatment	-	25 (2/8)
Day 0	2	100 (8/8)
Day 0	8	100 (8/8)
Day 3	8	100 (8/8)
Day 4	8	63 (5/8)
Day 5	8	38 (3/8)

Groups of 4–5 week-old BALB/c mice were treated i.p. with WNIG (2 or 8 mg/mouse), 4 h, or on days 3, 4, or 5 after injection of 10–20 LD_50 _of WNV. Mice were observed daily for mortality for 21 days.

#### WNIG therapy of immunosuppressed mice

Resistance to viral infection can be modified by treatment with dexamethasone [21,26]. Seven to 8 weeks old mice were chosen for this experiment, since we found that such mice are more resistant to low dose WNV infection than are younger animals (data not shown). Mice were injected s.c with two doses of 60 μg/mouse of dexamethasone. The first dose was administered 2 h before infection with 5–10 PFU of WNV NY-99 strain. A second dose was administered 1 day later.

Dexamethasone treatment increased the susceptibility of adult mice to WNV, thereby lowering the survival rate from 58% to less than 19%. Treatment of dexamethasone-immunosuppressed mice with 0.2 or 1.0 mg WNIG 4 h after viral infection resulted in 100% survival (Table 4).

**Table 4:** Therapy of immunosuppressed mice by WNIG.

Treatment group	Survival % (live/treated)
WNV control	58 (7/12)
Dexamethasone	19 (3/16)^a^
Dex + WNIG (1.0 mg/mouse)	100 (17/17)
Dex + WNIG (0.2 mg/mouse)	100 (16/16)

Group of 7–8 week-old BALB/c mice received two s.c. injection of 60 μg/mouse, dexamethasone (Dex), 2 hours before, and one day after infection with WNV. Mice were treated i.p. with WNIG (1.0 or 0.2 mg/mouse), 4 h after infection with 5–10 PFU of WNV. Mice were observed daily for mortality for 21 days. No mortality was observed in no-infected dexamethasone-treated mice.

^a^p < 0.001, as compared to the WNIG (0.2–1.0 mg) group.

## Discussion

Antibody-based therapy against viral and bacterial infections has been successfully practiced since the end of the 19th century [27]. Since the emergence of West Nile virus (WNV) as an important pathogen in a number of regions, including the continental USA, the potential use of specific antibodies for protection and treatment of infections associated with WNV has been studied in several animal models, including different mouse strains and Syrian golden hamsters [13,18,28-30].

We have previously shown, using a mouse model, that commercial preparations of IVIG from normal Israeli blood donors imparts protection to mice against viral infection. The emergence of WNV in many regions of the continental USA after 1999 has allowed others to confirm the efficacy in animal models, of IVIG produced from specific US regions [19]. Using a different approach, humanized monoclonal antibodies against specific epitopes in the viral envelope showing high *in-vivo *potency were developed [31]. Despite these successes it is still easier to develop a new formulation derived from a known pharmaceutical product with an excellent safety profile, such as IVIG [1]. Therefore, the use of IVIG with high WNV antibody levels is considered a leading approach for treatment of WNV infection. Although the potential use of IVIG from regions endemic for WNV has been was reported, a serious limiting factor in applying this concept is the relatively low levels of specific antibodies and very high batch-to-batch variance in WNV antibody levels, especially in IVIGs produced in countries such as the USA where the exposure of donors to WNV is limited and not homogenous [19]. Even in hyper-endemic regions such as Israel, only a fraction of the population has a history of exposure to WNV [17].

Our finding that only 8–10% of the plasma units from the normal donor population contributed almost all of the anti-WNV antibodies to the plasma pools allowed the development of a highly effective WNV treatment option The IVIG produced from positive WNV antibody-containing plasma units selected by ELISA, termed WNIG, showed promising potential in a cell neutralizing assay, with about 10-fold higher anti-WNV antibody levels being detected, as compared to regular IVIG-IL (9900 compared to 1032 WNU, by PRNT_50_, and 7608 compared to 668 AU, by ELISA, respectively). Indeed, good correlation was shown between the ELISA and PRNT_50 _results. Tests in mice confirmed the *in vitro *analysis by showing 5–10 fold enhancement (per g of total IgG) of efficacy when compared to regular IVIG-IL produced from Israeli donors and at least one log increase compared to IVIG-US produced from US donors.

When considering WNIG as a practical clinical option, treatment of patients with active encephalitis should be evaluated. The penetration of antibodies across the blood brain barrier (BBB) is highly inefficient but it is speculated that during active disease, the BBB becomes partially permeable thereby allowing penetration of significant quantities of antibodies to the infected site. As the level of antibodies in the serum is directly correlated to antibody levels in the brain, the goal of any treatment is to supply as many antibodies as possible, especially when trying to combat active disease. The protective effects of IVIG and monoclonal antibodies against infection already established in the brain was previously shown in animal models [15,18,28]. In human, intravenous immunoglobulin treatment was reported to be associated with improvement and elimination of the signs and symptoms of WNV infection [16,17]. Our results support the superiority of WNIG over regular IVIG-IL when given to mice even 3 or 4 days after infection, at the point where WNV had already established infection in the brain [18,32]. Augmenting the level of antibodies against WNV may thus prove to be an important treatment strategy for infection, particularly for the elderly and those with immune systems deficiencies [33]. Immuno-compromised subjects represent a major target population for passive antibody treatment or prophylaxis against WNV infection. Concomitant administration of dexamethasone and pathogens leads to widespread suppression of the innate immune response [26], enhanced viral replication and mortality [21]. It was shown that a broad range, non-specific immunosuppression increases the sensitivity of golden hamsters to WNV infection [34]. Engle and Diamond [28] showed that antibodies can only partially restore the protection against WNV infection thus suggesting that an efficient anti-viral response requires the combined activities of the innate and specific immune systems. However, we show here that WNIG alone was highly effective even when co-administered with the aggressive immuno-suppression agent, dexamethasone, which affects the innate immunity [26]. Treatment with WNIG offered complete protection to mice injected with dexamethasone, while the mortality among control infected mice injected with dexamethasone exceeded 80%.

When considering the practical application of passive immunotherapy to fight WNV infection, the total quantity of specific antibodies administered is limited by the maximum quantity of IVIG allowed for IV infusion (up to 2 g/kg). The use of WNIG will allow the administration of 5–10 times more specific antibodies, as compared to regular IVIG-IL, thus providing significantly enhanced therapeutic potential.

## Conclusion

The protective efficacy of WNIG, when administered 3 and 4 days after infection, suggests that the use of IVIG containing a high titer anti-WNV antibodies offers great potential for controlling active infection, even in the CNS. The possibility of exploiting the augmented levels of specific antibodies in a small fraction of the population by selection of plasma units before processed into IVIG will allow for the use of acceptable doses of IgG in patients, thereby increasing their chances for survival or lowering the risks of immediate and/or long term adverse effects.

## Abbreviations

Ab: antibodies; DMEM: Dulbecco's modified eagle medium; FCS: fetal calf serum; i.p.: intraperitoneal; IVIG: intravenous immunoglobulin; IVIG-IL: IVIG prepared from plasma of Israeli blood donors; IVIG-US: IVIG prepared from plasma of US blood donors; LD50: lethal dose 50%; MEM: modified eagle medium; PFU: plaque forming units; s.c.: subcutaneous; WNIG: IVIG prepared from plasma of Israeli blood donors with anti-WNV antibodies; WNV: West Nile virus.

## Competing interests

DBN, OGY, IS, YK, AG, MS, AP and BRZ declare that they have no competing interests. NO, IN and OL are employees of OMRIX Biopharmaceuticals.

## Authors' contributions

DBN and NO were responsible for the animal studies design, results interpretation and drafting the manuscript; IN, OL and NO were involved in concept development, IVIG production and characterization and study design. OGY carried out the mouse ELISA assays under the supervision of AP. IS, YK and MS were involved in the animal study designs and had a critical technical contribution to the animal model. BRZ contributed in all aspects of the animal study design and data interpretation. All of the authors were involved in critical reading of the manuscript and read and approved the final manuscript.

## Pre-publication history

The pre-publication history for this paper can be accessed here:

http://www.biomedcentral.com/1471-2334/9/18/prepub
